# Comparative synaptosome imaging: a semi-quantitative method to obtain copy numbers for synaptic and neuronal proteins

**DOI:** 10.1038/s41598-018-33130-6

**Published:** 2018-10-04

**Authors:** Katharina N. Richter, Hanna Wildhagen, Martin S. Helm, Jan-Eike Ußling, Thomas Schikorski, Silvio O. Rizzoli

**Affiliations:** 1 University Medical Center Göttingen, Institute for Neuro- and Sensory Physiology, Humboldtallee 23, Göttingen, 37073 Germany; 2 Cluster of Excellence Nanoscale Microscopy and Molecular Physiology of the Brain, Göttingen, Germany; 3 International Max Planck Research School Molecular Biology, Göttingen, Germany; 4 Department of Neuroscience, Universidad Central del Caribe, Bayamon Puerto Rico, United States of America

## Abstract

Protein copy numbers can be measured by biochemical methods ranging from quantitative Western Blotting to several mass spectrometry approaches. Such methods only provide average copy numbers, obtained over large cell numbers. However, copy number estimates for single cells or single organelles could be obtained by combining biochemical characterizations with an imaging approach. We performed this here for synaptic proteins, in a protocol that we termed comparative synaptosome imaging for semi-quantitative copy numbers (CosiQuant). In brief, in CosiQuant we immunostain in parallel biochemically-characterized synaptosomes, for which we have already determined the average protein copy numbers, and the samples of interest (such as neuronal cultures). We then derive the copy numbers in the samples of interest by comparing the immunofluorescence intensities. We measured the intensities not only in arbitrary fluorescence units, but also as numbers of antibodies per synaptosome, for a large number of targets. This implies that other groups can immediately apply CosiQuant for these targets, by simply estimating the number of antibodies per structure of interest. CosiQuant should therefore be a useful addition to the growing set of imaging techniques for synaptic neuroscience.

## Introduction

The quantitative organization of neurons has been the subject of countless scientific studies during the past decades, with synapse physiology being one of the most studied areas of neuroscience^1–3^. The research focus has typically been on explaining the mechanisms of neuronal communication, and finally on understanding the causes for neurological disorders.

Such disorders generally produce slight but significant changes in the basic molecular anatomy of the neuron, meaning the spatial organization and copy numbers of proteins within the neuron. The information on protein copy numbers in compartments such as the synapse is especially important, as it enables us to identify potential bottlenecks of cellular and molecular mechanisms^4–6^. As an example, mapping the protein composition of the synaptic vesicle^7^ has shown that the vesicular proton pump is only present in 1–2 copies per vesicle, and is thus one of the most likely molecules to generate a bottleneck (a rate-limiting factor) in synaptic vesicle recycling mechanisms. As a second example, endocytosis cofactors have also been shown to be limiting in the vesicle recycling pathway, while the exocytosis-related proteins are far more abundant, and are unlikely to be limiting^8,9^. In addition, knowledge on absolute copy numbers is also desirable from a technical point of view, as it renders comparisons between independent studies far more precise.

Numerous methods have been therefore developed to determine protein copy numbers, mainly by biochemical means. These techniques comprise Quantitative Western Blotting^10^ and mass spectrometry (MS) approaches like iBAQ (intensity-based absolute quantification^11^) and AQUA (absolute quantification approach^12^). Quantitative Western Blotting relies on the comparison of SDS-PAGE band intensities between a sample of interest and purified recombinant variants of the protein of interest, run on the gels in known amounts^9^. In many mass spectrometry approaches the estimation of the abundance of particular proteins is accomplished by comparing the spectra of the analyzed proteins and/or peptides to a standard. In AQUA this standard comprises an isotopically labeled peptide that reproduces a region of the protein of interest^12^, and which is spiked into the analyzed sample. iBAQ is a label-free method, in which one spikes accurately quantified proteins in the samples of interest, followed by in-solution digestion and mass spectrometry analysis. The intensities of the different peptides identified are summed to generate the overall protein intensities (for each protein of interest), and these are correlated to the known spiked amounts. This enables the use of a relatively simple linear regression to identify the amounts of the native proteins of interest^11,13,14^.

In all of these approaches the samples are usually cell culture lysates or brain homogenates, which precludes the analysis of variations between single cells or cell regions. To account for this, methods combining mass spectrometry and an imaging technique have been developed, like MALDI-TOF-TOF (matrix-assisted laser desorption ionization coupled to a tandem time-of-flight analysis) imaging, which makes it possible to map the intensities of MS spectra to the corresponding position in a sample^15^. However, accurate protein quantification is difficult to achieve, and the spatial resolution is still too low (usually about 20–50 µm) to obtain sub-cellular information. Alternatively, approaches for protein quantification based purely on imaging techniques could, in principle, provide the resolution needed for comparisons of protein copy numbers between cells or sub-cellular regions. For example, one can express GFP-tagged proteins of interest in the sample, followed by immunostaining for the proteins of interest^16^. One can then obtain estimates for the copy numbers of native (wild-type) proteins present in each cellular location by comparing the immunostaining signals to an estimation of the GFP copy numbers. However, these techniques are often difficult, as both the GFP signals and the immunostainings need to be carefully calibrated, and can only be applied to samples where the expression of GFP-tagged proteins is efficient.

Here we take advantage of synaptosomes that we have previously characterized by biochemical methods^9^ to establish a relatively easy imaging-based method for the estimation of protein copy numbers in neurons: comparative synaptosome imaging for semi-quantitative copy numbers (CosiQuant). Our approach relies on immunostaining the samples of interest and these synaptosomes in parallel. We then compare the resulting intensities in a semi-automated fashion. Protein copy numbers in the sample of interest can then be inferred from the known values in the synaptosome preparations, for which we derived estimates for more than 1000 proteins^9^. We tested this technique on cultured hippocampal neurons, and found that it works well for the estimation of neuronal proteins in this system.

Furthermore, we generalized this method, by removing the need for other laboratories to compare directly fluorescence intensities in our biochemically-characterized synaptosomes and in their preparations of interest. We expressed the fluorescence intensities we measured in synaptosomes in the form of “average numbers of antibodies per synaptosome”. This implies that other laboratories can estimate the numbers of antibodies in their structures of interest, and can then compare them with the numbers we provide, which would enable the estimation of protein copy numbers. We were able to validate this approach by turning to older datasets from the laboratory, not collected for the purpose of this work, which thus served as an independent control.

## Results

**Figure 1:**
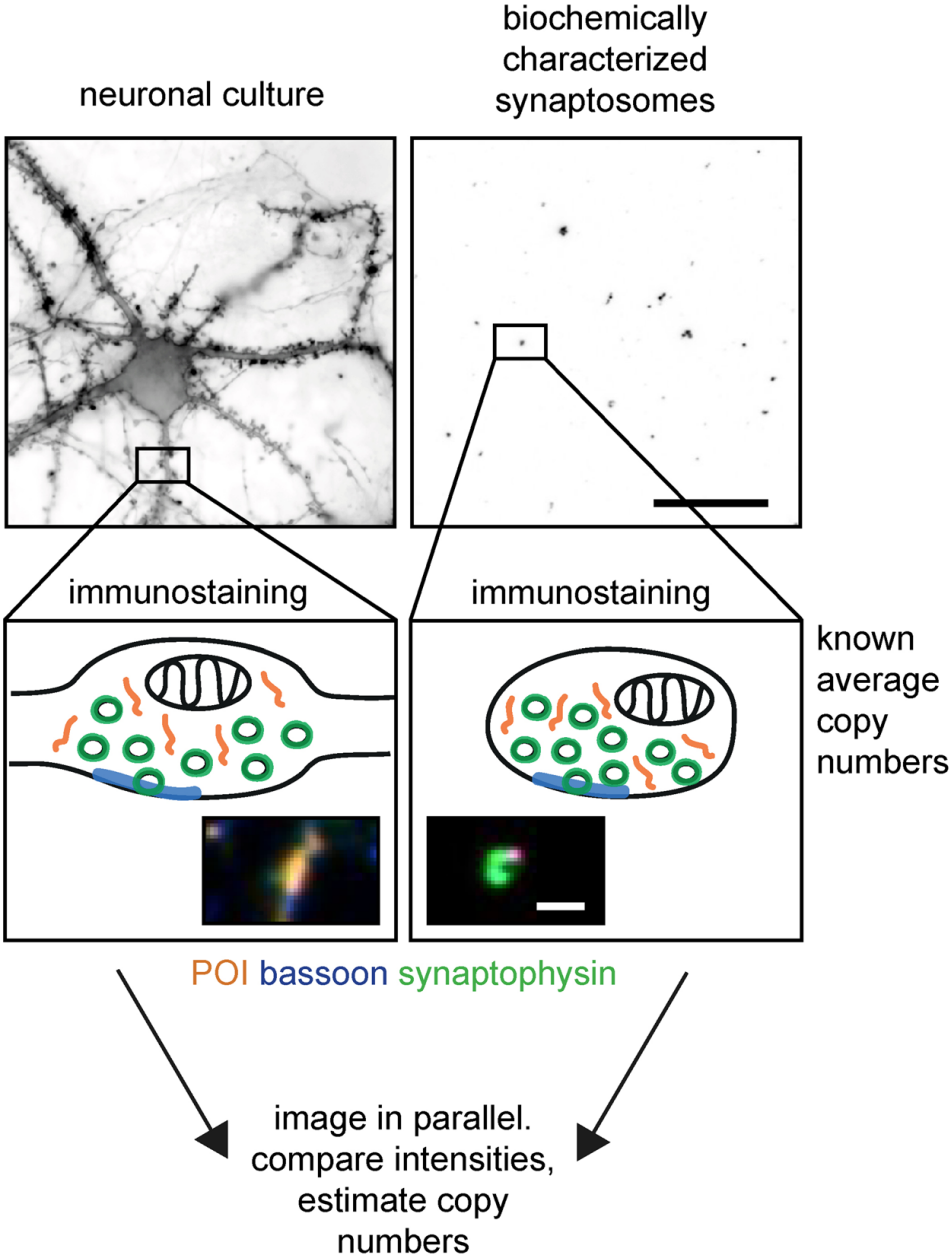
Principle of the comparative imaging approach (CosiQuant). Biochemically characterized synaptosome preparations are immobilized on coverslips. Cultured hippocampal neurons are immunostained in parallel with the synaptosomes for the synaptic vesicle marker synaptophysin (green), the active zone marker bassoon (blue) and the protein of interest (orange). The insets show example images of the fluorescently labeled structures (synapses/synaptosomes). Fluorescent signals for all labeled proteins are acquired, and intensities are compared between the synaptosome and neuron samples. Finally, protein copy numbers in cultured neuronal synapses can be estimated by comparing the intensities. Scale bar in overview images = 25 µm; scale bar in insets = 2 µm.

For an initial proof-of-principle, we tested the comparative imaging approach on a very well established system, the primary hippocampal neuron culture. The protocol works as follows (Fig. 1): frozen synaptosome preparations, which have been biochemically characterized in a previous study^9^, are thawed and are immobilized on coverslips. The synaptosomes and cultured hippocampal neurons are then immunostained in parallel for two synapse markers (synaptophysin for synaptic vesicles, and bassoon for the active zone) and for the protein of interest (POI). The fluorescent signals are acquired in the three separate channels, and the intensities of these signals are measured. The data are further processed, and afterwards signal intensities of the POI’s derived from both preparations are compared. Based on the knowledge about protein copy numbers in the synaptosome preparations, copy numbers of the POI in hippocampal neuron synapses can be interpolated.

We tested the comparative imaging approach for 10 synaptic markers: the synaptic vesicle marker synaptophysin, the active zone marker bassoon, as indicated above, followed by four additional synaptic vesicle proteins (the fusion SNARE VAMP2, the glutamate transporters vGlut1/2, the calcium sensor synaptotagmin 1, and the synaptophysin-related protein synaptogyrin), two plasma membrane SNAREs (SNAP25 and syntaxin 1), the soluble vesicle-binding protein synapsin 1/2, and the endosomal protein syntaxin 12/13 (see^3^ for details on the different proteins). We have previously derived copy numbers for all of these proteins in the synaptosome preparations^9^. At the same time, thoroughly characterized antibodies are available for immunostainings.

**Figure 2:**
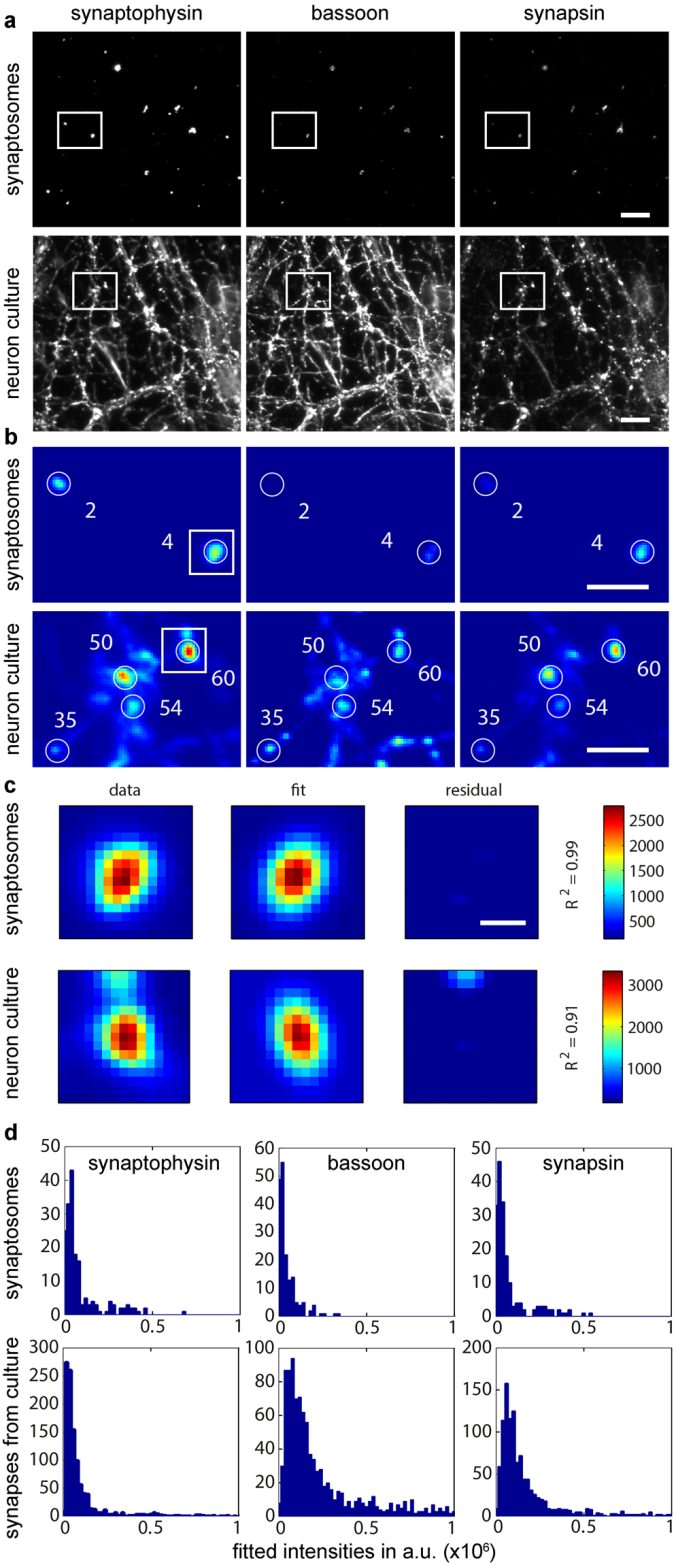
Image analysis for synapsin as protein of interest. (**a**) Example images of synaptosomes and cultured hippocampal neurons immunostained for synaptophysin, bassoon and synapsin. Scale bar = 10 µm (**b**) Automatic synapse selection by a custom-written Matlab routine in the areas indicated in the images in a. Synapse detection is based on the signal intensities of the synaptophysin staining (first channel). The selected synapses are numbered so fitted parameters (e.g. intensity) can later be assigned to the respective signals. Scale bar = 5 µm (**c**) Example of the Gaussian fits for the synapses indicated in panel b. The first image shows the original intensity data (scale in arbitrary units on the far right), the second image shows the model that is fitted to the data, and the third image indicates the residuals, i.e. the deviations of the data from the 2D Gaussian distribution. The R^2^ values of each fit are indicated on the right. Images are scaled in pixel. Scale bar = 800 nm (5 pixels) (**d**) Intensity histograms of all fitted synapses for the three channels (prior to final filtering).

Example images for the immunostainings are shown in Fig. 2a, with synapsin 1/2 as the POI. The images, comprised of the three channels for synaptophysin, bassoon and the POI, were analyzed by a custom-written Matlab routine, as described in Methods. In a first step, potential synapses in each image were detected based on the local intensity maxima in the synaptophysin (synapse marker) signal (Fig. 2b). Application of an initial intensity threshold ensures that a huge fraction of the image noise is not taken into account. At the same time, the co-presence of the bassoon signal ensures that the detected objects are indeed synapses, and not only synaptophysin protein assemblies or vesicles transported along the neurites. For each synapse candidate the size, intensity and position are determined using a Gaussian fit (Fig. 2c). The R^2^ value for each fit is calculated, and is used in a filtering step later on. Subsequently, the intensities of all three channels for all detected synapses are plotted (Fig. 2d). This data set includes many synapses that are not fitted correctly, or might not represent real/single synapses. Thus, a final filtering step has been included in the data analysis, which excludes synapse candidates with a fit worse than a set R^2^ threshold. This results in a data set containing information about the size, position and intensities of synapses, carefully pruned to represent real synapses.

**Figure 3:**
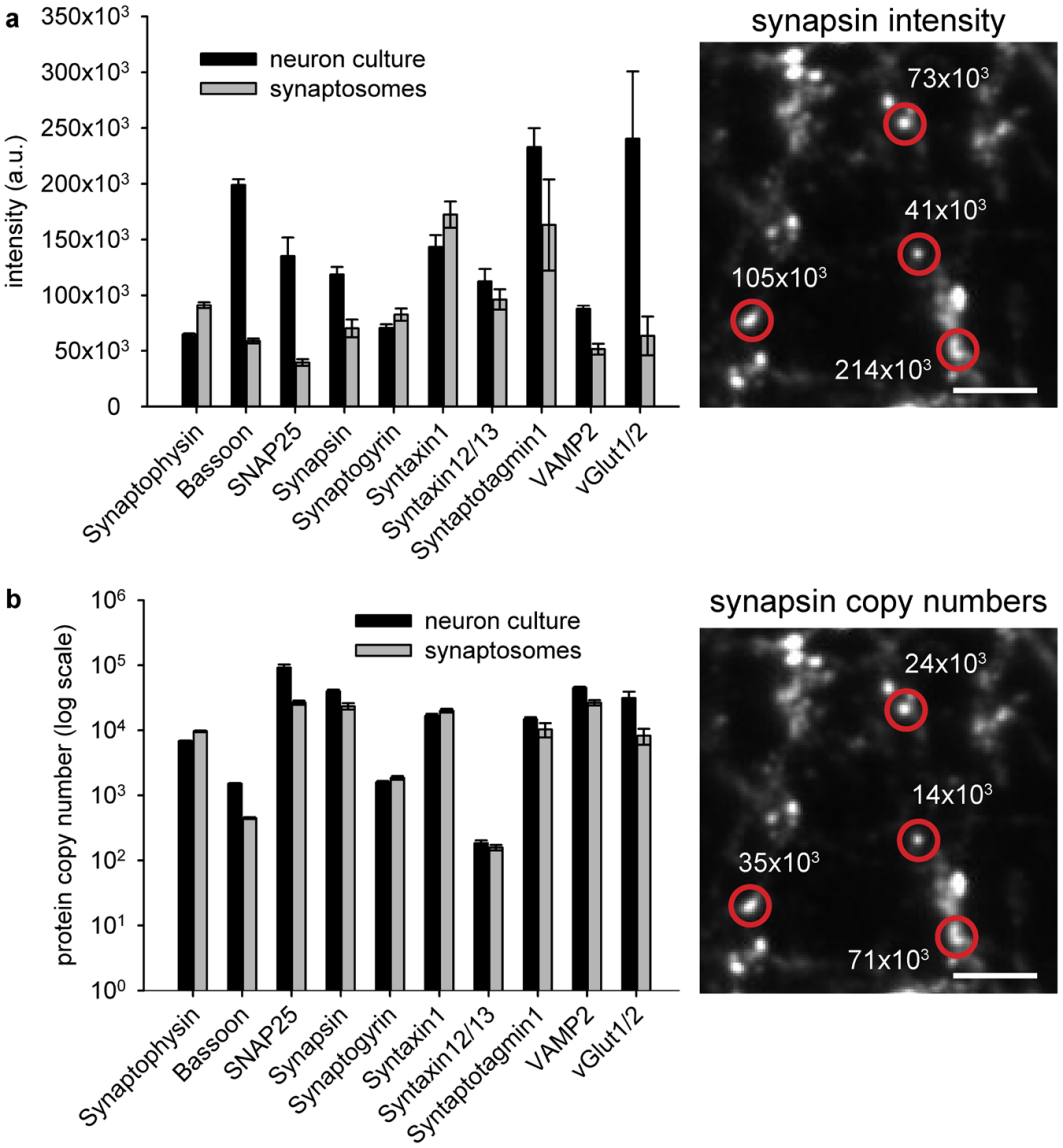
Comparison of intensities derived from labeled proteins in synaptosomes and neuronal synapses. (**a**) Absolute intensities (in arbitrary units) of the 10 labeled proteins in synaptosome preparations and cultured hippocampal neurons, expressed as mean values derived from all selected synapses (after final filtering). The right panel shows example synapses, marked with the respective intensities for the synapsin 1/2 stainings (in arbitrary units). Scale bar = 5 µm. (**b**) Average synaptic protein copy numbers, obtained by dividing the synapse intensities by the average synaptosome intensities, and multiplying with the average copy numbers per synaptosome from^9^. Example synapses with the respective synapsin 1/2 copy numbers are shown in the right panel. Scale bar = 5 µm. In contrast to the graph in (**a**), the graph in (**b**) is shown in log scale. Bar graphs represent mean values with standard errors of the mean (SEM). N = 875 synaptosomes (for synaptophysin and bassoon), 24–184 synaptosomes (for all other POI’s), 1809 cultured synapses (for synaptophysin and bassoon), and 51–354 synapses (for all other POI’s).

For further analysis we collected the measured intensities for all POIs, derived from all images. Mean intensities were calculated and were compared between stainings of synaptosomes and cultured neurons (Fig. 3a). We first investigated synaptophysin, which is the most specific marker of synaptic vesicles^7,10,17^, being found almost exclusively in vesicles, unlike other vesicle proteins that are often also found on the plasma membrane (for example synaptotagmin 1 or VAMP2^18,19^). The number of synaptic vesicles per synaptosome averages ~380^9^, measured in electron microscopy. In cultured hippocampal synapses only 250 ± 26 synaptic vesicles are present on average (mean ± SEM). This was calculated from 30 electron microscopy 3D reconstructions, with the following individual vesicle numbers: 36, 86, 89, 95, 119, 124, 128, 136, 137, 173, 185, 191, 197, 197, 212, 224, 233, 248, 249, 252, 257, 302, 315, 377, 403, 450, 509, 516, 526, 544. This analysis indicates that the synaptosomes contain on average 35% more synaptic vesicles. A similar value was found for the mean synaptophysin signal in our intensity comparison (29%), suggesting that these immunostaining signals are a reliable measure for the relative protein numbers in synaptosomes and hippocampal neurons.

**Figure 4:**
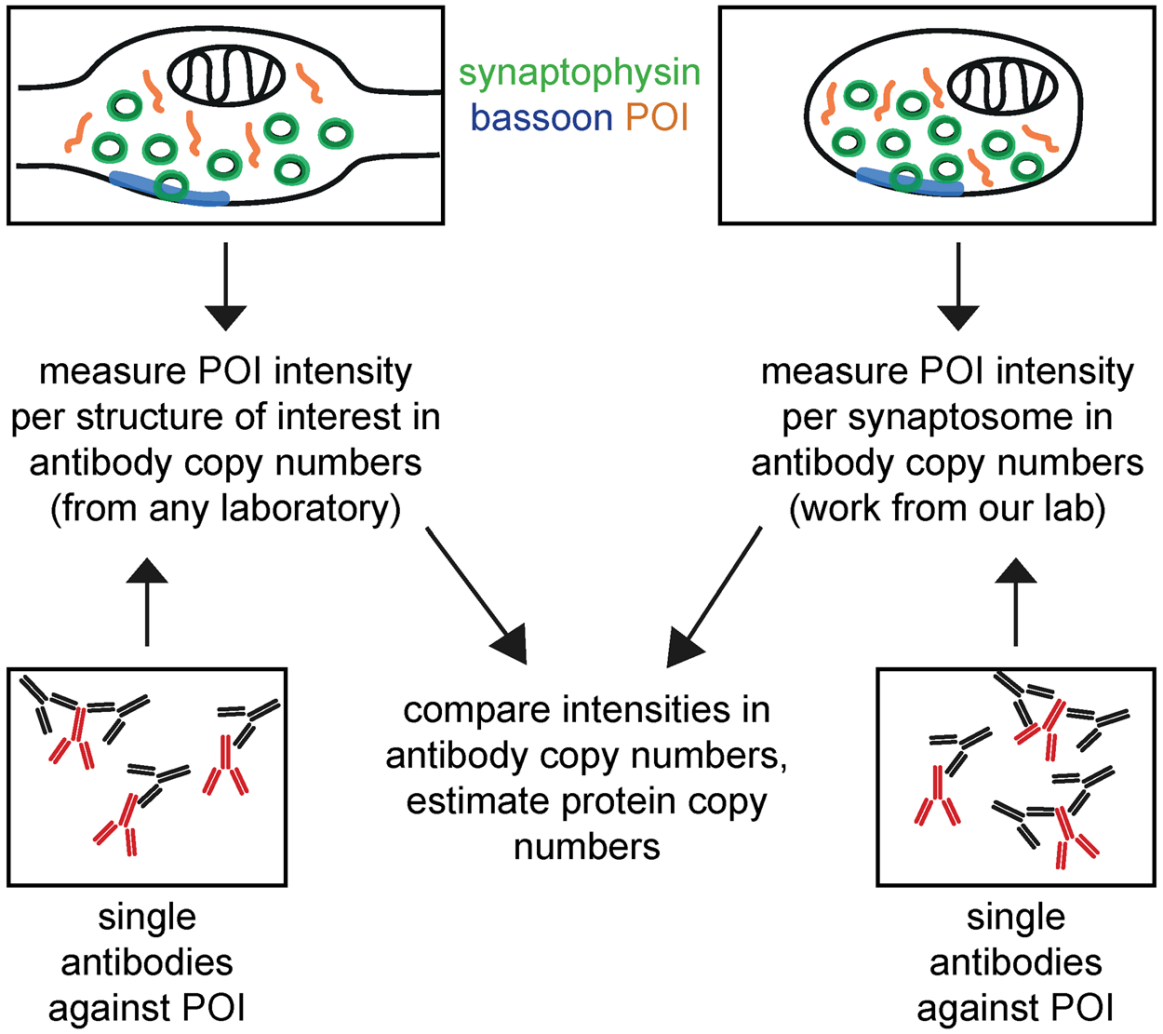
A comparative approach based on using the “number of antibodies per structure” as a measure for immunostaining intensities. The intensity of the immunolabaled synaptosomes and structure of interest (here: cultured neuronal synapses) can be measured (upper panels). Fluorescent intensities from single antibodies absorbed to a glass coverslip and immunolabeled with the same secondary antibodies as used for the synaptosomes/synapses can be determined in parallel (lower panels). This makes it possible to express the intensity of labeled synaptosomes and structures of interest in terms of antibody copy numbers. This has been done by our laboratory for over 100 target proteins in synaptosomes, and can be done easily for any structure of interest by any other laboratory. Finally, intensities can be compared in terms of antibody copy numbers, and protein copy numbers can be calculated from this.

Conclusions about the copy numbers of the other investigated proteins can now be drawn in a similar fashion, and absolute copy numbers can be estimated by comparisons with protein copy numbers from^9^, by dividing the fluorescence intensities of the neuronal synapses by the average synaptosome intensity, followed by multiplying the result with the (published) average copy numbers per synaptosome (Fig. 3b). Nevertheless, this is only useful if one can use the exact same synaptosome preparations analyzed by Wilhelm and colleagues^9^ for the comparative staining experiments. Since this is not the case for other laboratories, we also provide here the number of single antibodies (used for the immunostainings) per synaptosome for over 100 different targets (Tables 1 and 2). This will enable the comparison to immunostainings of any sample of choice performed with the same antibody, without the need for immunolabeling again the synaptosomes. This procedure only requires one to immunostain the protein of interest in the sample of choice, and to determine the number of single antibodies per structure of interest (Fig. 4). The number of antibodies can then be compared between synaptosomes (Tables 1 and 2) and the sample of choice, and thus protein copy numbers can be estimated.

**Table 1:** Numbers of primary antibodies per synaptosome for over 100 targets.

Target protein	Avergae # of ab’s	SEM/ROV	Average # of protein copies*	SEM	Company	Cat number	Species	Dilution
Actin	23.615	1.817 (SEM)	22074.6	1909.62	Novus Biologicals	NB600-535	mouse monoclonal	1:500
ADAM22	20.238	0.864 (SEM)	***		Novus Biologicals	NBP2-22425	mouse monoclonal	1:500
AKT (PKB)	10.326	0.239 (SEM)	***		Cell Signaling	4691	rabbit monoclonal	1:400
Alpha-SNAP	16.216	1.475 (SEM)	1150.7	46.62	Synaptic Systems	112 111	mouse monoclonal	1:200
Amphiphysin	19.001	1.973 (SEM)	1194.2	60.04	Synaptic Systems	120 002	rabbit polyclonal	1:500
AP180	15.174	1.139 (SEM)	3736.4	207.63	Synaptic Systems	155 003	rabbit polyclonal	1:500
AP2	19.136	1.546 (SEM)	2324.7	81.99	Abcam	ab75995	rabbit monoclonal	1:500
APP	14.186	2.776 (SEM)	6283.6	584.51	Millipore	MAB348	mouse monoclonal	1:100
Arc	18.419	0.545 (SEM)	***		Synaptic Systems	156 003	rabbit polyclonal	1:1000
BACE1	17.660	0.886 (SEM)	115.84	2.75	Santa Cruz	M-83	rabbit polyclonal	1:100
Bassoon	60.331	6.221 (ROV)	446.14	37.71	Enzo	ADI-VAM-PS003-F	mouse monoclonal	1:100
Bassoon	27.538	2.626 (ROV)	446.14	37.71	Synaptic Systems	141 002	rabbit polyclonal	1:500
Calbindin	33.403	0.917 (ROV)	296.88	13.22	Swant	CB38a	rabbit polyclonal	1:500
Calcineurin	11.718	0.188 (SEM)	138.67 (isoform B)	91.82	Synaptic Systems	387 002	rabbit polyclonal	1:1000
Calmodulin	27.019	4.493 (SEM)	8659.9	445.47	Novus Biologicals	NB110-55649	rabbit monoclonal	1:500
Calmodulin	13.510	1.0193 (ROV)	8659.9	445.47	Abcam	ab45689	rabbit monoclonal	1:100
Calretinin	16.688	2.724 (ROV)	369.24	2.47	Swant	CR7699/4	rabbit polyclonal	1:500
CAPS1	46.108	12.0256 (ROV)	196.42	28.58	Abcam	ab32014	mouse monoclonal	1:1000
CAPS1	23.302	2.013 (SEM)	196.42	28.58	Abcam	ab69797	rabbit polyclonal	1:500
CB1 (Anti-Cannabinoid Receptor)	12.842	0.745 (ROV)	***		Abcam	ab23703	rabbit polyclonal	1:50
CDC42	13.380	0.479 (SEM)	800.4	268.073	Thermo Scientific	PA1-092	rabbit polyclonal	1:100
ChromograninA	8.852	0.276 (ROV)	Presumably similar to ChromograninB		Synaptic Systems	259 003	rabbit polyclonal	1:500
ChromograninB	13.082	5.006 (ROV)	15.67	4.19	Synaptic Systems	259 103	rabbit polyclonal	1:500
ChromograninC	11.123	0.8085 (ROV)	Presumably similar to ChromograninB		Abcam	ab12241	rabbit polyclonal	1:250
Clathrin Heavy Chain	37.678	5.6 (SEM)	3472.47	174.65	BD Biosciences	610499	mouse monoclonal	1:500
Clathrin Light Chain	13.465	1.154 (ROV)	4554.06	296.74	Synaptic Systems	113 011	mouse monoclonal	1:500
Complexin1/2	15.858	2 (SEM)	2488.2	149.49	Synaptic Systems	122 002	rabbit polyclonal	1:500
CSP	16.542	3.041 (SEM)	941.18	48.86	Synaptic Systems	154 003	rabbit polyclonal	1:500
DLGAP1	31.073	4.709 (ROV)	52.961	27.765	Novus Biologicals	NBP1-76911	rabbit polyclonal	1:50
Doc2A/B	26.170	5.973 (ROV)	3696.5	164.19	Synaptic Systems	174 203	rabbit polyclonal	1:500
Dopamine ReceptorD1	16.985	1.9254 (ROV)	***		Abcam	ab40653	rabbit polyclonal	1:1000
Dopamine ReceptorD2	20.186	0.418 (SEM)	***		Merck	AB5084P	rabbit polyclonal	1:500
Drebrin1	22.188	0.532 (SEM)	342.075	85.238	Novus Biologicals	NB100-1951	mouse monoclonal	1:100
Dynamin1/2/3	17.379	1.382 (SEM)	2326.4	83.87	Synaptic Systems	115 002	rabbit polyclonal	1:500
EndophilinI	12.681	1.999 (SEM)	2524.4 (all isoforms)	67.27	Synaptic Systems	159 002	rabbit polyclonal	1:500
Epsin1	20.954	1.306 (SEM)	92.88	4.3	Novus Biologicals	EPR3023	rabbit polyclonal	1:100
ERp72 (PDIA4)	17.546	0.585 (SEM)	***		Cell Signaling	5033	rabbit monoclonal	1:100
GluK1 (Kainate Receptor)	15.725	2.207 (ROV)	***		Alomone	AGC-008	rabbit polyclonal	1:100
GluN1 (Kainate Receptor)	15.382	2.161 (SEM)	***		Synaptic Systems	114 011	mouse monoclonal	1:1000
GluN2A (Glutamate Receptor)	23.966	1.091 (SEM)	***		NeuroMab	75–288	mouse monoclonal	1:100
GluN2B (Glutamate Receptor)	8.549	1.06 (SEM)	83.6224	20.6121	NeuroMab	75–101	mouse monoclonal	1:100
GluR3 (Glutamate Receptor)	20.061	0.998 (SEM)	***		Invitrogen/Life Technologies	32–0400	mouse monoclonal	1:100
Homer1	9.363	1.947 (SEM)	712.817	83.691	Synaptic Systems	160 011	mouse monoclonal	1:500
Homer3	12.718	0.691 (ROV)	207.483	66.367	Synaptic Systems	160 303	rabbit polyclonal	1:250
Hsc70	24.154	3.72 (SEM)	8210.1	404.5	Santa Cruz	sc7298	mouse monoclonal	1:500
IGF-1R	10.494	0.935 (ROV)	***		Cell Signaling	3027	rabbit polyclonal	1:300
Intersectin1	26.077	2.955 (SEM)	3096.5	277.62	Haucke (Berlin)		rabbit polyclonal	1:500
Kir2.1	8.312	0.3 (ROV)	***		Novus Biologicals	NBP1-95482	rabbit monoclonal	1:100
Kv1.1	13.047	4.988 (ROV)	201.2956	95.671	Thermo Scientific	PA5-19593	rabbit polyclonal	1:100
Kv2.1	10.647	1.674 (ROV)	***		Synaptic Systems	231 002	rabbit polyclonal	1:500
Munc13a	19.037	1.047 (SEM)	1551.3	53.18	Synaptic Systems	126 102	rabbit polyclonal	1:500
Munc18a	21.108	1.53 (SEM)	4253.4	207.07	Synaptic Systems	116 002	rabbit polyclonal	1:500
Myosin5a	9.245	1.271 (SEM)	157.24	20	Sigma-Aldrich	M5062	rabbit polyclonal	1:200
NavBeta1	18.611	1.972 (SEM)	830.8294	248.9188	Alomone	ASC-041	rabbit polyclonal	1:50
nAChRBeta2	21.164	0.718 (SEM)	***		Alomone	ABC-012	rabbit polyclonal	1:100
NaKATPase	29.913	0.961 (SEM)	3771.7	481.7	Thermo Scientific	MA3-915	mouse monoclonal	1:1000
Nav1.1	13.934	1.261 (SEM)	***		Merck	06-811	rabbit polyclonal	1:100
Nav1.3	10.685	0.805 (SEM)	***		Alomone	ASC-004	rabbit polyclonal	1:250
nNOS	7.441	0.773 (ROV)	***		Thermo Scientific	PA1-033	rabbit polyclonal	1:100
NSF	15.662	0.432 (SEM)	677.45	213.02	Synaptic Systems	123 002	rabbit polyclonal	1:100
NSF	17.220	0.976 (SEM)	677.45	213.02	Synaptic Systems	123 002	rabbit polyclonal	1:500
Parvalbumin	33.327	0.435 (ROV)	681.1	34.31	Swant	PV25	rabbit polyclonal	1:500
Piccolo	32.092	0.322 (ROV)	100.45	8.4	Synaptic Systems	142 003	rabbit polyclonal	1:200
PSD95	17.742	1.163 (SEM)	1500.2	349.9	Sigma	P246	mouse monoclonal	1:200
PSD95	19.390	1.387 (SEM)	1500.2	349.9	Cell Signaling	3450	rabbit monoclonal	1:100
Rab3a	19.254	2.613 (SEM)	18846.58	996.01	Synaptic Systems	107 003	rabbit polyclonal	1:500
Rab5a	29.144	4.168 (SEM)	633.62	37.26	Cell Signaling	3547 S	rabbit polyclonal	1:500
Rab7a	21.976	2.29 (SEM)	4457.2	319.8	Santa Cruz	sc81922	rabbit polyclonal	1:100
Rab9	10.173	1.552 (ROV)	***		Cell Signaling	5118	rabbit polyclonal	1:100
Rapsn	15.000	1.443 (SEM)	***		Atlas Antibodies	HPA039475	rabbit polyclonal	1:100
Rim1	22.028	1.419 (ROV)	38.63	4.23	Synaptic Systems	140 003	rabbit polyclonal	1:200
SCAMP1	34.862	2.961 (SEM)	1459.5	115.53	Synaptic Systems	121 002	rabbit polyclonal	1:100
Sec22b	15.399	9.38 (ROV)	118.69	38.72	Synaptic Systems	186 003	rabbit polyclonal	1:100
Septin5	24.894	1.786 (SEM)	1726.2	64.38	Haucke (Berlin)		rabbit polyclonal	1:500
Septin7	14.637	0.168 (SEM)	2320.5	98.66	Atlas Antibodies	HPA029524	rabbit polyclonal	1:50
Shank1	10.116	0.515 (SEM)	141.276	35.11	Synaptic Systems	162 013	rabbit polyclonal	1:500
Shank2	15.660	0.638 (ROV)	168.2534	12.6241	Synaptic Systems	162 202	rabbit polyclonal	1:500
Shank3	22.504	3.638 (ROV)	181.28	53.24	Synaptic Systems	162 302	rabbit polyclonal	1:500
SNAP23	12.955	0.997 (SEM)	265.61	17.75	Synaptic Systems	111 202	rabbit polyclonal	1:100
SNAP25	10.415	2.71 (ROV)	26686.08	5287.39	Synaptic Systems	111 011	mouse monoclonal	1:100
SNAP29	19.561	2.654 (SEM)	77.47	6.47	Synaptic Systems	111 302	rabbit polyclonal	1:500
SNAP29	7.413	0.79 (ROV)	77.47	6.47	Synaptic Systems	111 302	rabbit polyclonal	1:500
SNAP47	9.079	0.546 (ROV)	~200**		Synaptic Systems	111 403	rabbit polyclonal	1:200
Stargazin	5.314	0.296 (SEM)	143.78	12.33	Alomone	ACC-012	rabbit polyclonal	1:200
SV2A/B	16.201	1.63 (SEM)	4616.65	128.17	Jahn Department		mouse monoclonal	1:100
Synapsin1/2	21.991	3.462 (SEM)	23422.77	1300.03	Synaptic Systems	106 002	rabbit polyclonal	1:500
Synaptogyrin	56.224	2.57 (SEM)	1854.8	110.49	Synaptic Systems	103 002	rabbit polyclonal	1:100
Synaptojanin	19.497	0.712 (SEM)	365.61	40.31	Synaptic Systems	145 003	rabbit polyclonal	1:100
Synaptotagmin1	15.737	1.671 (SEM)	10332	1079.2	Synaptic Systems	105 102	rabbit polyclonal	1:1000
Synaptotagmin2	15.451	1.823 (SEM)	297.28	11.37	Synaptic Systems	105 123	rabbit polyclonal	1:100
Synaptotagmin4	9.333	0.795 (ROV)	***		Synaptic Systems	105 143	rabbit polyclonal	1:1000
Synaptotagmin5/9	18.341	0.318 (SEM)	***		Synaptic Systems	105 053	rabbit polyclonal	1:100
Synaptotagmin7	29.852	1.613 (SEM)	182.64	3.54	Synaptic Systems	105 173	rabbit polyclonal	1:100
Synaptotagmin7	7.239	1.007 (ROV)	182.64	3.54	Synaptic Systems	105 173	rabbit polyclonal	1:250
Syndapin (Pacsin)	11.612	0.493 (SEM)	3201	131.28	Synaptic Systems	196 002	rabbit polyclonal	1:500
SynGAP1	7.941	1.278 (ROV)	622.07	90.73	Thermo Scientific	PA1-046	rabbit polyclonal	1:1000
Syntaxin1	16.991	1.826 (SEM)	20096	999.43	Synaptic Systems	110 011	mouse monoclonal	1:100
Syntaxin13	13.925	0.842 (SEM)	157.83	3.49	Synaptic Systems	110 131	mouse monoclonal	1:100
Syntaxin16	25.428	1.991 (SEM)	91.27	5.68	Synaptic Systems	110 162	rabbit polyclonal	1:100
Syntaxin16	12.975	0.172 (ROV)	91.27	5.68	Synaptic Systems	110 162	rabbit polyclonal	1:100
Syntaxin2	12.214	2.445 (SEM)	~100–200**		Synaptic Systems	110 022	rabbit polyclonal	1:100
Syntaxin3	11.433	4.084 (ROV)	<100**		Synaptic Systems	110 033	rabbit polyclonal	1:100
Syntaxin4	14.329	5.247 (ROV)	~100-200**		Synaptic Systems	110 042	rabbit polyclonal	1:100
Syntaxin5	17.280	0.66 (ROV)	~100–200**		Synaptic Systems	110 053	rabbit polyclonal	1:100
Syntaxin6	28.117	3.863 (SEM)	121.67	8.96	BD Biosciences	610636	mouse monoclonal	1:500
Syntaxin7	55.981	21.623 (ROV)	78.6	4.45	Jahn Department		mouse monoclonal 109.1	1:100
Syntaxin8	13.045	5.342 (ROV)	~100–200**		Synaptic Systems	110 083	rabbit polyclonal	1:100
TfR	21.293	0.719 (SEM)	***		Abcam	ab84036	rabbit polyclonal	1:100
TOM20	13.826	0.379 (SEM)	528.81	155.68	Sigma-Aldrich	WH0009804M1	mouse monoclonal	1:200
Tubulin	21.351	1.367 (SEM)	12056	615.3	Synaptic Systems	302 203	rabbit polyclonal	1:3000
VAMP1	17.671	1.315 (ROV)	3884.3	181.95	Synaptic Systems	104 002	rabbit polyclonal	1:500
VAMP2	21.158	1.199 (SEM)	26448	661.62	Synaptic Systems	104 211	mouse monoclonal	1:500
VAMP2	29.226	1.068 (SEM)	26448	661.62	Synaptic Systems	104 211	mouse monoclonal	1:1000
VAMP4	15.872	0.792 (SEM)	100.59	10.03	Synaptic Systems	136 002	rabbit polyclonal	1:100
VAMP7	8.528	0.924 (SEM)	~100–200**		Abcam	ab68776	rabbit polyclonal	1:100
vATPase	129.622	10.131 (SEM)	742.37	32.97	Synaptic Systems	109 002	rabbit polyclonal	1:100
VDAC1	19.532	1.269 (SEM)	14422.99	720.71	Santa Cruz	sc32063	rabbit polyclonal	1:100
Vglut1/2	13.518	1.565 (SEM)	8254.1	224.3	Synaptic Systems	135 503	rabbit polyclonal	1:100
Vti1a	12.390	0.487 (SEM)	50.55	2.51	BD Biosciences	611220	mouse monoclonal	1:100
Vti1a	17.383	5.03 (SEM)	50.55	2.51	BD Biosciences	611220	mouse monoclonal	1:100

Average numbers of antibodies per synaptosome and respective information about the conditions of immunostainings from which the numbers have been derived. The values are mean values obtained either from typically several hundreds of analyzed synapses or several experiments, which were done by multiple investigators. Column C shows either the standard error of the mean (SEM; in case of averaged synapses) or the range of values (ROV; in case of averaged experiments).

*Based on Wilhelm *et al*.^9^.

**Numbers estimated by the authors based on the copy numbers of functionally-related SNAREs, taking into account the high correlation between SNARE numbers in different pathways, as observed in the original work cited here.

***Not determined in the original work (Wilhelm *et al*.^9^), but currently under measurement in the Rizzoli laboratory for future references.

**Table 2:** Numbers of primary antibodies per synaptosome for over 100 targets (continued).

Fixation	Blocking	Permeabilization
4% PFA	2.5% BSA	0.1% Triton X-100
4% PFA	2.5% BSA	0.3% Tween 20
3% Glyoxal	2.5% BSA	0.3% Tween 20
4% PFA	2.5% BSA	0.1% Triton X-100
4% PFA	2.5% BSA	0.1% Triton X-100
4% PFA	2.5% BSA	0.1% Triton X-100
4% PFA	2.5% BSA	0.1% Triton X-100
4% PFA	2.5% BSA	0.1% Triton X-100
3% Glyoxal	2.5% BSA	0.3% Tween 20
4% PFA	2.5% BSA	0.1% Triton X-100
4% PFA	2.5% BSA	0.1% Triton X-100
4% PFA	2.5% BSA	0.1% Triton X-100
4% PFA	2.5% BSA	0.1% Triton X-100
3% Glyoxal	2.5% BSA	0.3% Tween 20
4% PFA	2.5% BSA	0.1% Triton X-100
3% Glyoxal	2.5% BSA	0.3% Tween 20
4% PFA	2.5% BSA	0.1% Triton X-100
4% PFA	2.5% BSA	0.1% Triton X-100
3% Glyoxal	2.5% BSA	0.3% Tween 20
3% Glyoxal	2.5% BSA	0.3% Tween 20
3% Glyoxal	2.5% BSA	0.3% Tween 20
3% Glyoxal	2.5% BSA	0.3% Tween 20
3% Glyoxal	2.5% BSA	0.3% Tween 20
3% Glyoxal	2.5% BSA	0.3% Tween 20
4% PFA	2.5% BSA	0.1% Triton X-100
4% PFA	2.5% BSA	0.1% Triton X-100
4% PFA	2.5% BSA	0.1% Triton X-100
4% PFA	2.5% BSA	0.1% Triton X-100
4% PFA	2.5% BSA	0.3% Tween 20
4% PFA	2.5% BSA	0.1% Triton X-100
3% Glyoxal	2.5% BSA	0.3% Tween 20
3% Glyoxal	2.5% BSA	0.3% Tween 20
3% Glyoxal	2.5% BSA	0.3% Tween 20
4% PFA	2.5% BSA	0.1% Triton X-100
4% PFA	2.5% BSA	0.1% Triton X-100
4% PFA	2.5% BSA	0.1% Triton X-100
4% PFA	2.5% BSA	0.3% Tween 20
3% Glyoxal	2.5% BSA	0.3% Tween 20
3% Glyoxal	2.5% BSA	0.3% Tween 20
3% Glyoxal	2.5% BSA	0.3% Tween 20
4% PFA	2.5% BSA	0.3% Tween 20
3% Glyoxal	2.5% BSA	0.3% Tween 20
3% Glyoxal	2.5% BSA	0.3% Tween 20
3% Glyoxal	2.5% BSA	0.3% Tween 20
4% PFA	2.5% BSA	0.1% Triton X-100
4% PFA	2.5% BSA	0.3% Tween 20
4% PFA	2.5% BSA	0.1% Triton X-100
3% Glyoxal	2.5% BSA	0.3% Tween 20
3% Glyoxal	2.5% BSA	0.3% Tween 20
3% Glyoxal	2.5% BSA	0.3% Tween 20
4% PFA	2.5% BSA	0.1% Triton X-100
4% PFA	2.5% BSA	0.1% Triton X-100
3% Glyoxal	2.5% BSA	0.3% Tween 20
4% PFA	2.5% BSA	0.3% Tween 20
3% Glyoxal	2.5% BSA	0.3% Tween 20
4% PFA	2.5% BSA	0.3% Tween 20
4% PFA	2.5% BSA	0.3% Tween 20
4% PFA	2.5% BSA	0.3% Tween 20
3% Glyoxal	2.5% BSA	0.3% Tween 20
4% PFA	2.5% BSA	0.3% Tween 20
3% Glyoxal	2.5% BSA	0.3% Tween 20
4% PFA	2.5% BSA	0.1% Triton X-100
4% PFA	2.5% BSA	0.1% Triton X-100
4% PFA	2.5% BSA	0.1% Triton X-100
3% Glyoxal	2.5% BSA	0.3% Tween 20
4% PFA	2.5% BSA	0.1% Triton X-100
4% PFA	2.5% BSA	0.1% Triton X-100
4% PFA	2.5% BSA	0.1% Triton X-100
3% Glyoxal	2.5% BSA	0.3% Tween 20
3% Glyoxal	2.5% BSA	0.3% Tween 20
4% PFA	2.5% BSA	0.1% Triton X-100
4% PFA	2.5% BSA	0.1% Triton X-100
3% Glyoxal	2.5% BSA	0.3% Tween 20
4% PFA	2.5% BSA	0.1% Triton X-100
3% Glyoxal	2.5% BSA	0.3% Tween 20
3% Glyoxal	2.5% BSA	0.3% Tween 20
3% Glyoxal	2.5% BSA	0.3% Tween 20
3% Glyoxal	2.5% BSA	0.3% Tween 20
4% PFA	2.5% BSA	0.1% Triton X-100
3% Glyoxal	2.5% BSA	0.3% Tween 20
4% PFA	2.5% BSA	0.1% Triton X-100
3% Glyoxal	2.5% BSA	0.3% Tween 20
3% Glyoxal	2.5% BSA	0.3% Tween 20
4% PFA	2.5% BSA	0.3% Tween 20
4% PFA	2.5% BSA	0.1% Triton X-100
4% PFA	2.5% BSA	0.1% Triton X-100
4% PFA	2.5% BSA	0.1% Triton X-100
4% PFA	2.5% BSA	0.1% Triton X-100
4% PFA	2.5% BSA	0.1% Triton X-100
4% PFA	2.5% BSA	0.1% Triton X-100
3% Glyoxal	2.5% BSA	0.3% Tween 20
3% Glyoxal	2.5% BSA	0.3% Tween 20
4% PFA	2.5% BSA	0.1% Triton X-100
3% Glyoxal	2.5% BSA	0.3% Tween 20
4% PFA	2.5% BSA	0.1% Triton X-100
3% Glyoxal	2.5% BSA	0.3% Tween 20
4% PFA	2.5% BSA	0.1% Triton X-100
4% PFA	2.5% BSA	0.1% Triton X-100
4% PFA	2.5% BSA	0.1% Triton X-100
3% Glyoxal	2.5% BSA	0.3% Tween 20
3% Glyoxal	2.5% BSA	0.3% Tween 20
3% Glyoxal	2.5% BSA	0.3% Tween 20
3% Glyoxal	2.5% BSA	0.3% Tween 20
3% Glyoxal	2.5% BSA	0.3% Tween 20
4% PFA	2.5% BSA	0.1% Triton X-100
4% PFA	2.5% BSA	0.3% Tween 20
3% Glyoxal	2.5% BSA	0.3% Tween 20
3% Glyoxal	2.5% BSA	0.3% Tween 20
3% Glyoxal	2.5% BSA	0.3% Tween 20
4% PFA	2.5% BSA	0.3% Tween 20
3% Glyoxal	2.5% BSA	0.3% Tween 20
4% PFA	2.5% BSA	0.1% Triton X-100
3% Glyoxal	2.5% BSA	0.3% Tween 20
4% PFA	2.5% BSA	0.1% Triton X-100
3% Glyoxal	2.5% BSA	0.3% Tween 20
4% PFA	2.5% BSA	0.1% Triton X-100
4% PFA	2.5% BSA	0.1% Triton X-100
4% PFA	2.5% BSA	0.1% Triton X-100
3% Glyoxal	2.5% BSA	0.3% Tween 20
4% PFA	2.5% BSA	0.1% Triton X-100

Additional information about the conditions of immunostainings.

**Figure 5:**
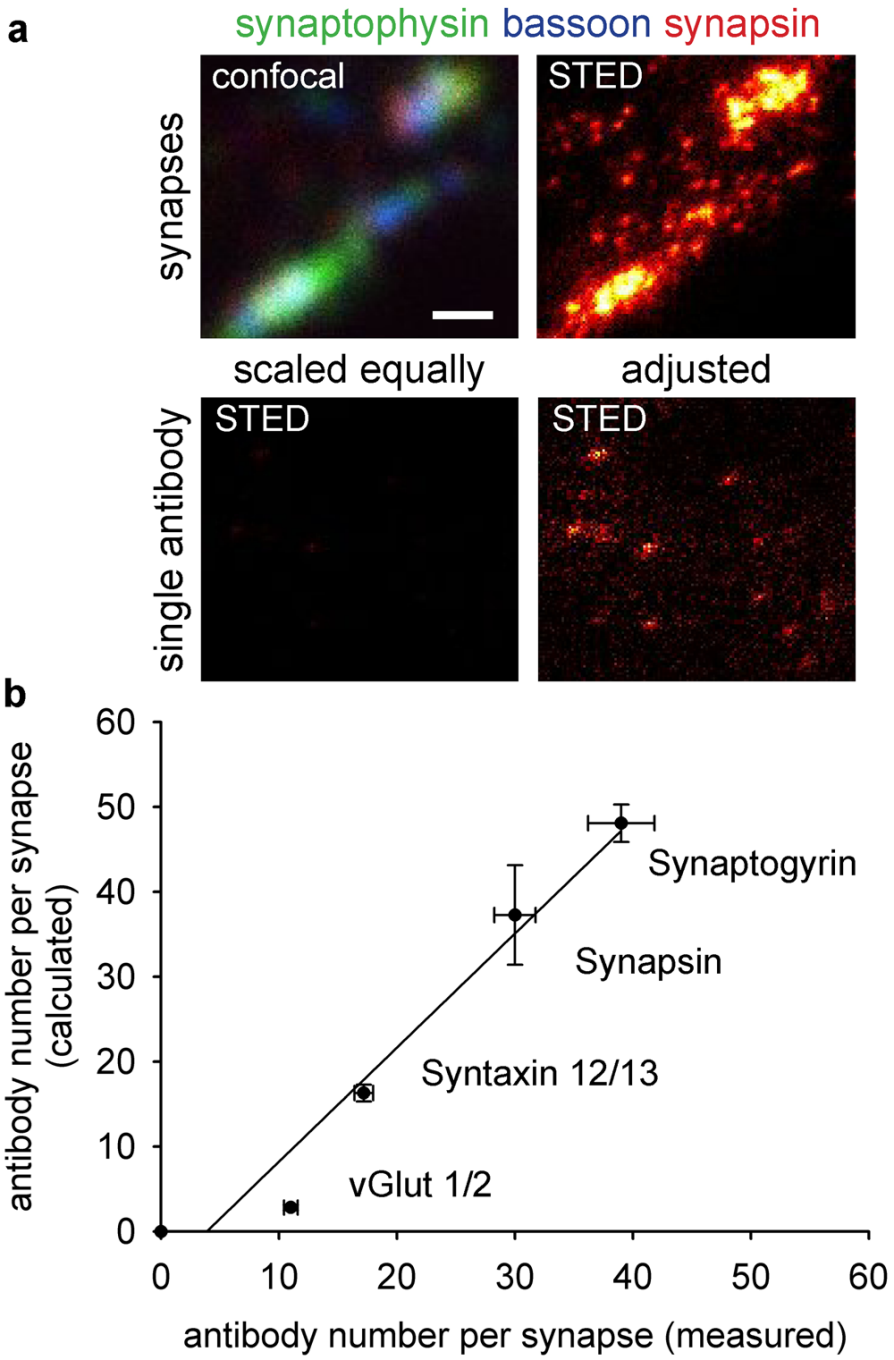
Numbers of single antibodies as a measure for relative immunostaining intensity (**a**) Confocal and STED images of hippocampal neuron synapses and single antibodies bound to a glass coverslip. Synapses were stained for synaptophysin, bassoon and synapsin1/2 (confocal image). Super-resolution images of the signal derived from the anti-synapsin antibodies were taken in synapses (top right) or in a single-antibody configuration (bottom panels; left: scaled identically to synapses; right: autoscaled to visualize the signal of single antibodies). Scale bar = 500 nm (**b**) 2-dimensional scatter plot comparing numbers of antibodies per synapse derived from the measurement described in a), along the Y axis, or derived by calculation from synapse/synaptosome ratio (taken from results in Fig. 3), along the X axis. The spots represent mean values, and error bars represent the standard error of the mean (SEM). N = 632, 660, 1034 and 1009 synapses for synapsin, synaptogyrin, syntaxin 12/13 and vGlut 1/2 respectively, from two independent experiments, for the measured data. The estimated data are extracted from Fig. 3, and therefore have the same N values. The black line represents a linear regression with a slope of 1.34 and R^2^ of 0.955.

In order to validate this approach, we tested it on older data from our laboratory, which were not generated for this purpose, but were available from experiments performed previously by different investigators, using the same antibodies at different points in time, between 2012 and 2014. We analyzed super-resolution images (acquired using STED microscopy) of hippocampal neuron cultures immunostained for 4 proteins, which we had also investigated previously in the comparative imaging experiment showcased in Fig. 3 (synapsin, synaptogyrin, syntaxin12/13 and vGlut1/2). The intensities of single antibodies spotted on coverslips (derived from the same samples) were measured (Fig. 5a), and the number of single antibodies per neuronal synapse was calculated by dividing the total intensity in a synapse by that of the single antibodies. This provides a direct, measured number of antibodies per synapse in culture, from experiments performed between 2012 and 2014. This is the type of measurement any laboratory would be able to obtain directly, in immunostaining experiments.

At the same time, we estimated the number of antibodies per synapse from our parallel immunostaining experiments, all performed in 2018. We simply multiplied the number of antibodies per synaptosome (from Tables 1 and 2) with the ratio between the synaptosome and the synapse intensities, obtained from the parallel experiments from Fig. 3. This estimate should be relatively precise. We then compared the overall results in a 2-dimensional scatter plot (Fig. 5b). The high correlation between the two methods indicates that the number of single antibodies per synapse is a reliable substitute for the relative intensity of an immunostaining. Therefore, numbers of antibodies per synaptosome provided by us in Tables 1 and 2 can be used for the comparative imaging approach. This makes CosiQuant an easily applicable method for the estimation of protein copy numbers, with sub-cellular resolution.

## Discussion

We introduced here an imaging-based method for the estimation of protein copy numbers, which can be implemented relatively easy in every lab equipped for standard immunohistochemistry experiments. CosiQuant relies on the comparison of immunolabeling signals from the sample of interest and biochemically characterized synaptosome preparations. In brief, we derive the protein copy numbers for POIs in samples of interest by comparing the immunostaining signals to those derived from synaptosome preparations with known copy numbers. Furthermore, we provide a substitute for this comparison (Figs 4 and 5), so that other laboratories can apply the comparative imaging method to their sample of interest without having to use the exact same synaptosome preparations as used here.

In contrast to methods like Quantitative Western Blotting or mass spectrometry, no extensive purification steps are needed, and a much higher spatial resolution can be achieved. Furthermore, the use of synaptosomes instead of other standards, such as purified proteins or fluorescent beads, provides a more realistic comparison to neuronal samples. Effects such as the orientation of different proteins within membranes or molecular complexes, protein density, or steric hindrances, which all affect antibody binding, will apply in an identical fashion to both cultured synapses and synaptosomes, thus reducing the experimental differences between the standard sample and the sample to be measured.

Nevertheless, the comparative imaging approach also exhibits several limitations. A major one concerns the fact that CosiQuant assumes that the synaptosomes and the sample of interest are immunostained equally well. This should not be a major concern regarding the immunostaining of synaptosomes and of cultured neurons, since factors like penetration of antibodies should be comparable in both of these thin samples. For samples like whole brains or organs, however, this might differ substantially (although we found this not to be the case under optimal immunostaining conditions, for ~20 different antibodies^9^). For such samples discrepancies in antibody penetration could be tested by staining for a GFP-tagged variant, whose fluorescence is used as a standard, and the differences in staining efficiency could be corrected for. Another procedure to ensure relatively equal staining conditions while comparing synaptosomes and whole brain/organ samples would be to prepare and to immunostain thin sections of the samples. In this fashion the sample depth can be defined, and can be limited to ~10 µm. In order to achieve this, one can prepare cryosections of freshly dissected samples. Brains or organs can be either snap-frozen or chemically fixed first and then frozen in liquid nitrogen or on dry ice. Subsequently, the samples can be cut on a cryostat into 10 µm thin sections, which can be immunostained on coverslips or glass slides.

Another limitation concerns the usage of numbers of single antibodies as a substitute to staining intensity for the synaptosome preparations. We provide here the numbers of single antibodies per synaptosome (Tables 1 and 2) for over 100 different antibodies. But in order to use these numbers for the comparison of samples, one needs to stain the sample of interest under the exact same conditions (including fixation, antibody concentration, or blocking conditions) as the synaptosomes. These conditions might not be applicable to all samples. Furthermore, CosiQuant cannot be used for target proteins that have not been biochemically characterized yet in our synaptosomes. Nevertheless, the initial database of antibody numbers per synaptosome we provide here may already prove useful, since it covers pathways and networks ranging from the cytoskeleton to synaptic vesicle exo- and endocytosis, or to the postsynaptic density. Another limitation concerns the fact that antibody staining intensities may not sum linearly, and thus some estimates may not be correct. It has been our experience that this type of error is largely negligible, at least for structures that are not immensely bright. For example, immunostained single antibodies, which should be identified by ~3 secondary antibodies, are indeed ~3 fold brighter than individual secondary antibodies^20,21^, both in STED microscopy and in epifluorescence microscopy. This may not necessarily hold true in every imaging system, and should be tested carefully.

In summary, CosiQuant provides a comparably easy method for the estimation of protein numbers, and should in principle be applicable to a variety of different samples. This should be especially relevant in samples that are not purifiable (as cells from the sensory system or the peripheral nervous system^22,23^) and thus cannot be analyzed by methods as mass spectrometry, which require abundant, purified samples.

## Methods

### Synaptosome preparation

Synaptosomes were purified by simple differential centrifugation and ficoll gradient centrifugation, as described previously in^9^. The synaptosome preparations from that study were snap frozen and stored at −80 °C. The frozen synaptosomes were used by thawing them on ice and immobilization on glass coverslips (for details see immunocytochemistry section).

### Primary hippocampal neuron culture

Rat primary hippocampal neuron cultures were prepared from newborn rats as described before^24,25^ and were cultured under standard conditions. Cultured neurons of 10 to 15 days *in vitro* (21 or 22 DIV for the calculation of single antibody numbers per synapse; Fig. 5 and Tables 1, 2) were used for immunocytochemistry experiments.

### Immunocytochemistry of synaptosomes and primary hippocampal neurons

For synaptosome immobilization on glass coverslips, the coverslips were coated with 5% bovine serum albumin (BSA, AppliChem #A1391,0500) over night at 4 °C. Frozen synaptosome preparations were thawed on ice and dilutions (in PBS) were spun down in a centrifuge (VWR MegaStar3.0R) for 40 min at 4000 rpm, at 4°. Fixation of synaptosomes and cultured neurons was done with a 4% PFA solution for 15 min at 4 °C and another 45 min at room temperature. Subsequently, preparations were washed briefly in PBS and quenched for 30 min in 100 mM NH_4_Cl. Permeabilization and background epitope blocking were achieved by 30 min incubation in PBS containing 0.1% Triton X-100 and 5% BSA (blocking solution). Both samples were incubated with primary antibodies diluted in blocking solution (prepared as one master mix) for 60 min at room temperature. After washing for 30 min in PBS the preparations were incubated with secondary antibodies, diluted in blocking solution for 60 min at room temperature. Primary and secondary antibodies used for all stainings, and the respective dilutions from a 1 mg/ml stock can be found in Table 3. Final washing in high-salt PBS (containing 500 mM NaCl) and PBS was followed by embedding in Mowiol. The samples were imaged with an epifluorescent Nikon microscope.

**Table 3:** Antibodies used for the immunostaining of cultured neurons and synaptosomes.

Target protein	Species	Company	Dilution
**primary antibodies**			
Synaptophysin	guinea pig	SySy (#101004)	1:300
Bassoon	mouse	Enzo (ADI-VAM-PS003-F)	1:100
SNAP25	rabbit	SySy (#111002)	1:500
Synapsin	rabbit	SySy (#106002)	1:500
Synaptogyrin	rabbit	SySy (#103002)	1:200
Syntaxin1	rabbit	SySy (#110302)	1:100
Syntaxin12/13	rabbit	SySy (#110133)	1:200
Synaptotagmin1	rabbit	SySy (#105102)	1:100
VAMP2	rabbit	SySy (#104202)	1:500
vGlut1/2	rabbit	SySy (#135503)	1:100
**secondary antibodies**			
anti-guinea pig IgG (Alexa488)	donkey	Dianova (#706-545-148]	1:100
anti-mouse IgG (Cy5)	donkey	Dianova (#715-175-150)	1:100
anti-rabbit IgG (Cy3)	donkey	Dianova (#711-165-152)	1:100

Some immunostainings of cultured neurons and synaptosomes used for calculating the number of single antibodies per synapse/synaptosome (Fig. 5 and Tables 1, 2) were performed with slight variations to the protocol described above. Neurons cultured in the Banker arrangement (locally separated from the astrocyte feeder layer^26^) were fixed with a 3% glyoxal solution^27^ and the blocking and permeabilization was achieved with 2.5% BSA and 0.3% Tween in PBS. Secondary antibody incubation was done for 60 min with Cy3-labeled donkey anti-mouse or rabbit Fab fragments (Dianova, #715-166-150 and #711-166-152) and Atto647N-labeled goat anti-mouse or rabbit antibodies (Rockland, #610-156-121 and #610-156-122). All other steps of the staining protocol remained the same.

The immunolabeling protocol for cultured neurons and synaptosomes stained for super resolution imaging (data used in Fig. 5 and Tables 1, 2) can be found in^9^.

Information about all antibodies used for stainings, in addition to the ones described in Table 3, can be found in Tables 1 and 2.

### Image acquisition

Comparative imaging of the immunolabled synaptosomes and cultured neurons was done with an inverted epifluorescence Nikon Eclipse Ti-E microscope. The microscope was equipped with an HBO 100 W lamp and images were acquired with an Andor IXON X3 897 camera or a Nikon DS-Qi2 camera (for images used to calculate synaptosome staining signals in terms of number of antibodies, Tables 1 and 2). The samples were imaged using a 100X PLAN APO oil immersion objective (NA 1.45). For multi-color imaging the following filter sets were used for Alexa488, Cy3 and Cy5 imaging: 470/40 nm (excitation, Alexa488), 525/50 nm (emission, Alexa488), 545/25 nm (excitation, Cy3), 605/70 nm (emission, Cy3), 620/60 nm (excitation, Cy5), 700/75 nm (emission, Cy5). Image acquisition software used was NiS-Elements AR (Nikon) and imaging parameters were kept the same for samples that were compared.

Super-resolution imaging (STED) and confocal imaging of neurons and synaptosomes (data used in Fig. 5 and Tables 1, 2) were performed with a Leica TCS SP5 STED microscope, exactly as described in^9^.

### Image analysis

The image analysis was a two-step process carried out via custom-written Matlab (The Mathworks Inc.) routines. In the first step, the aim was to obtain initial guesses for the positions of the synapses. For this purpose, the script searches for the local intensity maxima in the images immunostained for synaptophysin (synapse marker). In order to avoid too many false positives caused by intensity noise, the images were filtered before searching for the local maxima using a Gaussian kernel with standard deviation σ = 480 nm. In addition, intensity thresholds were applied, *i.e*. spots with very small peak intensities, which are most likely due to noise, were not taken into account. The thresholds were carefully chosen by eye, and they were set low enough so that real synapses were not accidentally removed. Thus at this point, due to the low thresholds, the set of selected synapses included a fraction of false positive synapse candidates. The set of synapse candidates was further filtered at a later stage of the image analysis.

In the second step, the script goes back to the raw (unfiltered) images and fits the exact positions, sizes and intensities for all synapse candidates using the initial guesses as obtained in the first step. For the fit a square region of interest (2.2 μm × 2.2 µm) around each candidate is defined, and all candidates in all channels are fitted, *i.e*. those stained for synaptophysin, bassoon and the respective protein of interest (POI). The fit model is a 2D Gaussian function of variable size, position, orientation, amplitude and offset. For further analysis, mainly the total (integrated) intensity of each synapse candidate was used. The R^2^ between the model and the data was calculated as a measure for the goodness of the fit.

In a final step, all fitted synapse candidates were filtered according to the R^2^ value for each channel (synaptophysin, bassoon, POI). Candidates with a R^2^ value below 0.85 (synaptophysin), 0.7 (bassoon) and 0.6 (POI) for the fit were discarded. Thus, sets of well fitted synapses and synaptosomes were left, which could be compared in terms of intensity.

For display purposes only (Figs 2a and 5) images were adjusted in brightness and contrast using ImageJ (Wayne Rasband, US National Institutes of Health). If intensities were compared, image adjustments in brightness and contrast were equally applied to all conditions.

### Data analysis

Intensities of the analyzed and filtered synapses and synaptosomes were collected and mean intensities were compared between neuron cultures and synaptosomes for each protein of interest. To analyze the signals in terms of antibody copy numbers, the intensities of the cellular structures (synaptosomes or cultured neurons) were divided by the average intensity of single antibodies immunostained on coverslips (thus equivalent to background antibody signals), as performed in the past^20^.

### Protocol for imaging single antibodies

A simple procedure enables the analysis of the intensities of single immunostained primary antibodies. We typically rely on coverslips coated with poly-L-lysine, which are incubated for 10–30 minutes with primary antibody dilutions (final concentrations of 10 µg/ml), in PBS. The coverslips are then fixed, using the same fixation buffer as desired in the final application (for example 4% PFA, with or without 0.1% glutaraldehyde, or 3% glyoxal; see^27^, for further details on different fixation protocols). After fixation a quenching procedure is performed for 15–30 minutes, with 50–100 mM NH_4_Cl in PBS, or 50–100 mM glycine in PBS. This is followed by 2–3 rapid washes with PBS.

Alternatively, the fixation step can be avoided completely, as in most experiments the primary antibodies are not subjected to fixation during the immunostaining procedure. We only perform the fixation procedure when analyzing antibodies that are normally taken up by living cells, and therefore are fixed during the immunostaining process.

The coverslips are then incubated for 15–30 minutes with PBS containing 2–3% BSA (blocking buffer). The blocking buffer coats the poly-L-lysine surface with BSA, and prevents the extensive binding of secondary antibodies to this surface. This is followed by incubating the coverslips with secondary antibodies (diluted to 10 µg/ml) in PBS containing 2–3% BSA, for 30–60 minutes. The coverslips are then washed extensively: 3 × 5 minutes with PBS containing high salt (500 mM NaCl), and 3 × 5 minutes with normal PBS (150 mM NaCl). The coverslips can then be mounted in the desired mounting medium, and can be imaged.

For the image analysis, we recommend applying a bandpass filter on the images, to detect the antibody spots, followed by Gaussian fits on the spots, to obtain the total signal intensity associated to each spot. The population of antibody intensities obtained should be fitted well by a single Gaussian, whose peak position indicates the average single primary antibody intensity. Performing this experiment with super-resolution is very convenient, since then large spots (full width at half maximum, FWHM, larger than 50 nm) can be discounted. They do not represent single antibodies, but presumably are caused by dirt on the coverslips.

Importantly, a very simple and practical application for obtaining this type of value, without any additional experiments, has been to investigate the background spots obtained on the clean coverslip areas adjacent to cultured neurons, in the normal immunostaining experiments used for determining protein intensities in the cultured neurons. It has been our experience that the results obtained are indistinguishable from those obtained when immunostaining antibodies on coverslips in separate experiments, as described above, provided that large spots, indicating dirt or cell debris on the coverslips, are discounted. Nevertheless, a number of experiments in which antibodies on coverslips are measured as indicated above should be performed, to test that the background spots from the cultures can be indeed trusted, since inappropriate handling of the cultures (poor fixation or blocking, for example) may result in the formation of extensive antibody clusters, which would perturb the measurements.

### Statistics

Bars and data points in Figs 3 and 5 show mean values. All error bars represent the standard error of the mean (SEM), calculated in Sigma Plot (Systat Software, Inc.), unless stated otherwise in the figure legend.

Column C in Table 1 shows the standard error of the mean (SEM) from typically several hundreds of analyzed synapses or the range of value (ROV) derived from several experiments, which were done by multiple investigators.

### Animals

Wild type Wistar rats (*Rattus norvegicus*) for the preparation of primary hippocampal neuron cultures and synaptosomes were obtained from the University Medical Center Göttingen. All animals were handled according to the specifications of the University of Göttingen and of the local authority, the State of Lower Saxony (Landesamt für Verbraucherschutz, LAVES, Braunschweig, Germany). All animal experiments were approved by the local authority, the Lower Saxony State Office for Consumer Protection and Food Safety (Niedersächsisches Landesamt für Verbraucherschutz und Lebensmittelsicherheit).

## Data Availability

The datasets generated during and/or analyzed during the current study are available from the corresponding author on reasonable request.
